# Characteristics and Pathways of Long-Stay Patients in High and Medium Secure Settings in England; A Secondary Publication From a Large Mixed-Methods Study

**DOI:** 10.3389/fpsyt.2018.00140

**Published:** 2018-04-16

**Authors:** Birgit A. Völlm, Rachel Edworthy, Nick Huband, Emily Talbot, Shazmin Majid, Jessica Holley, Vivek Furtado, Tim Weaver, Ruth McDonald, Conor Duggan

**Affiliations:** ^1^Division of Psychiatry and Applied Psychology, University of Nottingham, Nottingham, United Kingdom; ^2^Nottinghamshire Healthcare NHS Foundation Trust, Nottingham, United Kingdom; ^3^Department of Mental Health, Social Work and Integrative Medicine, Middlesex University, London, United Kingdom; ^4^Unit of Mental Health and Wellbeing, Warwick Medical School, University of Warwick, Coventry, United Kingdom; ^5^Birmingham and Solihull Mental Health NHS Foundation Trust, Birmingham, United Kingdom; ^6^Manchester Business School, University of Manchester, Manchester, United Kingdom

**Keywords:** forensic mental health services, length of stay, long-stay patients, mental health care, mentally disordered offenders, forensic psychiatry, hospitalization

## Abstract

**Background:** Many patients experience extended stays within forensic care, but the characteristics of long-stay patients are poorly understood.

**Aims:** To describe the characteristics of long-stay patients in high and medium secure settings in England.

**Method:** Detailed file reviews provided clinical, offending and risk data for a large representative sample of 401 forensic patients from 2 of the 3 high secure settings and from 23 of the 57 medium secure settings in England on 1 April 2013. The threshold for long-stay status was defined as 5 years in medium secure care or 10 years in high secure care, or 15 years in a combination of high and medium secure settings.

**Results:** 22% of patients in high security and 18% in medium security met the definition for “long-stay,” with 20% staying longer than 20 years. Of the long-stay sample, 58% were violent offenders (22% both sexual and violent), 27% had been convicted for violent or sexual offences whilst in an institutional setting, and 26% had committed a serious assault on staff in the last 5 years. The most prevalent diagnosis was schizophrenia (60%) followed by personality disorder (47%, predominantly antisocial and borderline types); 16% were categorised as having an intellectual disability. Overall, 7% of the long-stay sample had never been convicted of any offence, and 16.5% had no index offence prompting admission. Although some significant differences were found between the high and medium secure samples, there were more similarities than contrasts between these two levels of security. The treatment pathways of these long-stay patients involved multiple moves between settings. An unsuccessful referral to a setting of lower security was recorded over the last 5 years for 33% of the sample.

**Conclusions:** Long-stay patients accounted for one fifth of the forensic inpatient population in England in this representative sample. A significant proportion of this group remain unsettled. High levels of personality pathology and the risk of assaults on staff and others within the care setting are likely to impact on treatment and management. Further research into the treatment pathways of longer stay patients is warranted to understand the complex trajectories of this group.

## Introduction

For many forensic patients, hospitalization involves compulsory detention in a secure psychiatric unit with the aim of treating their mental disorder and offending behaviour whilst ensuring, as far as possible, the establishment of safety [1]. In the UK, patients are admitted to secure forensic services at low, medium, and high levels of therapeutic security because they have a history of serious violence and pose a serious or grave risk to the public [2], whether or not they have been formally convicted of an offence. The duration of such hospitalization is not time-limited, however, and in England length of stay in forensic psychiatric settings far exceeds that in general psychiatric services [3] and often also that of imprisonment for the same offence [4].

A substantial proportion of forensic patients in UK medium secure settings stay longer than the 2 years recommended for such units in early guidance [5, 6], and one study has suggested that as many as 27% of patients in both high and medium secure settings stay at least 10 years [7]. Reasons for delayed discharge from a secure forensic unit are likely to include poor response to treatment, ongoing safety issues, and lack of a suitable step-down facility. Concerns have been expressed that for some patients their stay in secure services is unnecessarily long, and frequently at an inappropriate level of security [8–11]. Whereas the concept of the long-stay forensic patient may be valid for those individuals who require life-long care [12], for others an inappropriately long hospital stay raises resource and ethical issues. This is because secure forensic services are expensive [13] and highly restrictive for those detained within them [14].

Forensic inpatient care in England differs from other countries in a number of complex ways, all of which can impact on a patient's length of stay in hospital. These arise from differences in the legal frameworks governing the detention of mentally disordered offenders [15], differences in the roles taken by health and justice authorities in deciding when and how forensic patients are transferred and discharged, different concepts of criminal responsibility and its role in determining admission to a forensic-psychiatric institution and the impact of prevailing sensitivities about perceived risk to others [16, 17]. A number of features of the English forensic care system are particularly relevant.

First, unlike in most other European countries, patients can be admitted to forensic-psychiatric services without having offended. Patients admitted to secure institutions in the UK without a formal offending history are often those who present with challenging behaviour in general psychiatric services, making it impossible to safely care for them there; these patients might have committed offences in institutions but they may not have led to prosecutions as the criminal justice agencies might not deem prosecution of patients who are already within an institution of sufficient public interest.

Second, patients in England and Wales are generally admitted to forensic care on the basis of clinical need at the time of sentencing (if an offender); absent or diminished criminal responsibility is not a criterion for admission as is the case in most other jurisdictions. The responsibility for decisions about transfer and discharge predominantly lies with the treating team (though in some cases the Ministry of Justice has to agree); the sentencing court plays no further role in decisions about the patient once admitted to hospital.

Third, inpatient forensic psychiatric care is available in England at three high secure hospitals, 57 medium secure units and, more recently, within a number of low secure facilities. Patients may be moved between hospitals of different levels of security, whereas in the Netherlands and in Germany, for example, different levels of security are provided within the same hospital; this potentially allows for easier transfer from one security level to another [16] which may facilitate throughput and result in shorter stays.

Fourth, individuals admitted to forensic-psychiatric care after having committed an offence may be held well beyond the time they would have been incarcerated had they received a prison sentence as a non-mentally-disordered individual [4]. In contrast, four countries within Europe (Croatia, Italy, Portugal, Spain) currently restrict the length of stay in forensic psychiatric care to the length of imprisonment a non-mentally disordered individual would have been sentenced to serve if convicted for the same offence [18], a process that can lead to shorter admissions.

Detention of psychiatric patients, both general and forensic, is regulated in the UK under the Mental Health Act 1984. Other than section 3 (a civil section, used for patients with no criminal conviction at the time of admission), there are two sections of the Act which are most commonly used for mentally disordered offenders: section 37 (initiated by a court of law at the time of sentencing, resulting in the patient being admitted to hospital instead of prison; readiness for discharge is decided by a senior doctor), or section 47 (transfer from prison for patients initially sentenced to imprisonment but then requiring care at some point during their sentence; the patient may return to prison at the end of treatment). Restrictions may be added so that the Ministry of Justice has to agree to conditional discharge from hospital or to transfer to another setting (section 41 for those on a section 37; or section 49 for those on a section 47). Inevitably, the need for Ministry of Justice approval for moves to other secure settings leads to delay in the transfer of patients and longer stays.

Comprehensive information is lacking on the numbers and characteristics of forensic patients in England that experience extended hospital stays. Previous research has identified a number of factors associated with longer stay populations, including severity of psychopathology, seriousness of offending, psychotic disorder, history of violence, substance misuse, non-engagement in interventions, and lack of step-down facilities [5, 12, 19–21], but has mostly been conducted in single secure units and has been based on discharge samples which neglects those who never achieve discharge. This study therefore aimed to provide a representative description of long-stay patients in high and medium secure settings in England. The main research question was: What are the characteristics of long-stay patients and the factors associated with long-stay and do they differ between high and medium secure settings?

## Materials and methods

### Sampling

Data on the characteristics of patients meeting the criteria for long-stay status were obtained from two of the three high secure hospitals and from a stratified sample of 23 of all 57 medium secure units in England at time of the study. Stratification was by sector (i.e., NHS or independent), geographical region, size and specialisation, with oversampling of units specialising in women and patients with intellectual disabilities. The medium secure sample comprised 14 units managed by the NHS and nine in the independent sector.

### Definition of “long-stay”

The threshold used to define long-stay status has been shown to vary widely between countries [18, 21] and there is currently no accepted definition of “long-stay.” In previous studies the point beyond which forensic inpatients have been considered as long-stayers has ranged from 2 to 15 years [21]. In the UK, thresholds of 8 [19] and 15 [22] years have been used in high secure samples, whereas for medium secure settings most studies have used a threshold of either 2 or 5 years. The 2-year threshold is in keeping with early guidance based on recommendations in the Butler report, published in 1975 [23], that medium secure units were intended to provide care for patients for whom there was a good prospect of discharge within 18 months to 2 years of admission [24]. However, more recent studies on length of stay in medium secure settings have shown that about 10–20% of patients stay for 5 years or longer and this threshold has also been used in two previous medium secure studies [3, 12]. Our piloting data from one high secure hospital suggested that just over 15% of patients stayed for 10 years or longer. We aimed to capture the more extreme end of long-stay and therefore a cut-off that captures 15–20% of the population seemed appropriate; this is also roughly the proportion of forensic patients residing in specific long-stay facilities in countries where such services exist. We therefore defined long-stay as 5 years in medium secure care, or 10 years in high secure care, or 15 years in continuous secure care if patients had stayed in a combination of high and medium secure settings. This defined a population large enough in size to provide meaningful conclusions for service developments but not so large that a substantial proportion of patients would be captured.

### Data collection and analysis

Data on length of stay were collected through medical records departments for all patients resident in participating units on 1 April 2013. Units identified their long-stay patients using the following procedure. First, they identified those whose stay in the current unit exceeded the defined threshold. Second, they identified those who had not stayed in their current units for a period exceeding our threshold but who were admitted from another high or medium secure setting; this was done to ascertain whether adding these spells of care led to the patient being identified as a long-stayer.

Data on characteristics of long-stay patients were obtained from detailed file reviews which were collected by unit staff to maintain anonymity. A number of measures were introduced in order to maximise consistency, including the development of a data collection proforma and training exercises in its completion. The training protocol included two exercises to assess understanding of the inclusion criteria and the documentation of criminal history. A pilot proforma was completed and reviewed by the study team with feedback given for all data collectors. Only if this seemed satisfactory were a further five proformas completed for review; full data collection began if sufficient quality of data collection was achieved. Units were paid administrative time for these tasks. Any queries or inconsistencies were fed back to the contact person in the appropriate unit for clarification.

Severity of offending history was estimated on a scale of 0–3 obtained by totalling the following: 1 point for age at first conviction <17 years; 1 point for more than six violent or sexual offences, or for a grave index offence where a discretionary or mandatory life sentence would have been available; 1 point for more than 15 non-violent/non-sexual offences. Psychiatric diagnoses according to ICD-10 were as recorded in each case file by the patient's consultant psychiatrist.

Quantitative data were analysed using Stata (version 13; StataCorp LP, College Station, TX, USA), and Statistical Product and Service Solutions software (version 21; IBM Corporation, Armonk, NY, USA). Descriptive statistics were calculated for medium and high secure samples separately. Categorical comparisons were made using cross-tabulation and chi-square tests. For continuous data, comparisons were made using *t*-tests, or Mann–Whitney non-parametric tests where variables deviated from an approximately normal distribution. A significance criterion of *p* < 0.05 and two-tailed tests were used throughout.

### Ethical considerations

This study was confined to data routinely collected by unit staff and transferred to the research team in a fully anonymised form; as such, it was deemed to constitute service evaluation by the sponsoring institution. Units were offered the option to exclude certain high-profile patients if they felt that data could not be provided in a way that would exclude incidental identification; one high secure unit excluded one patient under this procedure. The study was registered under Comprehensive Clinical Research Network Portfolio 129376, funded by the National Institute for Health Research and sponsored by Nottinghamshire Healthcare NHS Foundation Trust.

## Results

At the time of the study (1 April 2013), 116 (22.3%) of the 519 high secure patients and 285 (18.1%) of the 1572 medium secure patients met the long-stay criteria. Unless otherwise indicated, data presented below are therefore from a total sample of 401 long-stay patients.

### Sociodemographics

The majority of long-stay patients were single, male, with poor educational backgrounds and born in the UK (Table 1). Their mean age was 45 years. Two-thirds of the sample had no formal qualifications. Information on previous employment was available for 356 individuals of whom 23 had not been in the community beyond the age of 16 years. For the rest, when last in the community, 74% were unemployed. Overall, 39% had been in full- or part-time employment for a period of at least 6 months at some point in their lives.

**Table 1:** Sociodemographics of long-stay forensic patients.

	**Whole sample (*n* = 401)**	**High security (*n* = 116)**	**Medium security (*n* = 285)**	**Statistic**	***p*-value**
**GENDER**, ***n*** **(%)**					
Male	344 (85.8)	105 (90.5)	239 (83.9)	χ^2^ = 3.00	0.083
**AGE, YEARS**					
Mean (SD)	44.46 (11.26)	45.60 (9.76)	44.00 (11.79)	*t* = 1.29	0.197
Range	20–82	25–77	20–82		
**COUNTRY OF BIRTH (*****n*** = **397**^a^**)**, ***n*** **(%)**					
UK	364 (91.7)	107 (92.2)	257 (91.5)	χ^2^ = 0.07	0.797
Other	33 (8.3)	9 (7.8)	24 (8.5)	χ^2^ = 0.07	0.797
**RELATIONSHIP STATUS ON ADMISSION**, ***n*** **(%)**					
Married	11 (2.9)	4 (3.8)	7 (2.5)	χ^2^ = 0.44	0.506
In a relationship	1 (0.3)	0 (0)	1 (0.4)	χ^2^ = 0.38	0.537
Divorced or widowed	44 (11.4)	9 (8.5)	35 (12.5)	χ^2^ = 1.25	0.264
Never married	329 (85.5)	93 (87.7)	236 (84.6)	χ^2^ = 0.61	0.434
Total	385 (100.0)	106 (100.0)	279 (100.0)		
**EMPLOYMENT STATUS PRIOR TO ADMISSION (*****n*** = **333**^b^**)**, ***n*** **(%)**					
Full- or part-time employment	68 (20.4)	29 (35.0)	39 (15.6)	χ^2^ = 14.34	<0.001
Full- or part-time education	2 (0.6)	1 (1.2)	1 (0.4)	χ^2^ = 0.68	0.411
Unemployed	247 (74.2)	46 (55.4)	201 (80.4)	χ^2^ = 20.30	<0.001
Other	16 (4.8)	7 (8.4)	9 (3.6)	χ^2^ = 3.18	0.074
Total	333 (100.0)	83 (100.0)	250 (100.0)		
Ever in full- or part-time employment (*n* = 346^c^), *n* (%)	136 (39.3)	27 (31.4)	109 (41.9)	χ^2^ = 3.00	0.083
No formal qualifications (*n* = 365^d^), *n* (%)	241 (66.0)	62 (69.7)	179 (64.9)	χ^2^ = 0.69	0.405
Not in the community since 16 years (*n* = 356^e^), *n* (%)	23 (6.5)	16 (16.2)	7 (2.7)	χ^2^ = 21.36	<0.001
**CONTACT IN THE LAST 2 YEARS**, ***n*** **(%)**					
Family	260 (64.8)	76 (65.5)	184 (64.6)	χ^2^ = 0.03	0.856
Friends	21 (5.2)	4 (3.4)	17 (6.0)	χ^2^ = 1.05	0.305
Family & friends	73 (18.2)	19 (16.4)	54 (18.9)	χ^2^ = 0.37	0.546
Neither family nor friends	47 (11.7)	17 (14.7)	30 (10.5)	χ^2^ = 1.36	0.244

a *Country of birth unknown for 4 medium secure patients*.

b *Excludes those with no time in the community since 16 years of age*.

c *Data available from 86 high secure and 260 medium secure patients*.

d *Data available from 89 high secure and 276 medium secure patients*.

e *Data available from 99 high secure and 257 medium secure patients*.

No differences were observed between the high and medium secure samples of long-stay patients in terms of age, country of birth, marital status, or qualifications. However, significant differences were found in terms of their employment history; a lower proportion of the high secure sample than the medium secure patients (55 vs. 80%) were unemployed when last in the community, and significantly more individuals in the high secure sample had not been in the community since the age of 16 years (16 vs. 3%).

The majority of patients were currently in contact with either family members (65%) or friends (5%), or both (18%). The contact involved actual visits for the majority. Only 12% had not had any contact with either friends or family members in the past 2 years. There were no significant differences between the high and medium secure groups in terms of the proportion of patients having such outside contacts.

### Length of stay

For the long-stay sample, mean length of stay in continuous secure care was 175 months or 14.5 years, with about a fifth having stayed for longer than 20 years (Table 2). Median length of stay in the high secure sample was significantly longer than in the medium secure sample for continuous care as well as in the current unit. Ten patients from the high secure sample had been resident in secure care for longer than 30 years.

**Table 2:** Length of stay in continuous care for long-stay forensic patients.

	**Whole sample (*n* = 401)**	**High security (*n* = 116)**	**Medium security (*n* = 285)**	**Statistic**	***p*-value**
**LENGTH OF STAY, MONTHS**					
Mean (SD)	175.0 (103.9)	203.6 (86.2)	163.3 (108.3)		
Median (range)	155.2 (13.7–651.0)	183.6 (13.7–503.3)	128.4 (60.2–651.0)	*z* = 5.21	<0.001
**LENGTH OF STAY CATEGORIES**, ***n*** **(%)**					
5–10 years	144 (35.9)	7 (6.0)	137 (48.1)	χ^2^ = 63.30	<0.001
>10–20 years	178 (44.4)	86 (74.1)	92 (32.3)	χ^2^ = 58.51	<0.001
>20–30 years	53 (13.2)	13 (11.2)	40 (14.0)	χ^2^ = 0.58	0.448
>30 years	26 (6.5)	10 (8.6)	16 (5.6)	χ^2^ = 1.23	0.268

*Mann–Whitney non-parametric test (z) used where continuous variables deviated from an approximately normal distribution*.

### Pathways

Overall, 56% of the long-stay sample came to high or medium secure care from prison whereas 16% had been admitted from the community (Table 3). Regarding admission to their current unit, 47% had been admitted from medium secure care, while 24% had been admitted from high secure care and 20% from prison, with very low numbers admitted from other settings. Significantly more patients in the high secure sample were admitted to their current unit from prison compared to the medium secure sample.

**Table 3:** Admission source and moves between settings for long-stay forensic patients.

	**Whole sample (*n* = 401)**	**High security (*n* = 116)**	**Medium security (*n* = 285)**	**Statistic**	***p*-value**
**ADMISSION TO CONTINUOUS CARE**, ***n*** **(%)**					
Prison	225 (56.1)	69 (59.5)	156 (54.7)	χ^2^ = 0.75	0.385
Community	64 (16.0)	15 (12.9)	49 (17.2)	χ^2^ = 1.12	0.291
Other psychiatric setting	48 (12.0)	16 (13.8)	32 (11.2)	χ^2^ = 0.52	0.473
Low secure NHS	35 (8.7)	9 (7.8)	26 (9.1)	χ^2^ = 0.19	0.661
Low secure private	20 (5.0)	4 (3.4)	16 (5.6)	χ^2^ = 0.82	0.366
Other	9 (2.2)	3 (2.6)	6 (2.1)	χ^2^ = 0.09	0.768
**ADMISSION TO CURRENT UNIT**, ***n*** **(%)**					
High secure setting	97 (24.2)	23 (19.8)	74 (26.0)	χ^2^ = 1.69	0.193
Medium secure, private	117 (29.2)	13 (11.2)	104 (36.5)	χ^2^ = 25.51	<0.001
Medium secure, NHS	71 (17.7)	30 (25.9)	41 (14.4)	χ^2^ = 7.45	0.006
Prison	79 (19.7)	45 (38.8)	34 (11.9)	χ^2^ = 37.61	<0.001
Low secure, private	5 (1.2)	1 (0.9)	4 (1.4)	χ^2^ = 0.20	0.658
Low secure, NHS	15 (3.7)	1 (0.9)	14 (4.9)	χ^2^ = 3.76	0.053
Community	6 (1.5)	0	6 (2.1)	χ^2^ = 2.48	0.115
Other	11 (2.7)	3 (2.6)	8 (2.8)	χ^2^ = 0.02	0.902
**NUMBER OF UNIT MOVES DURING CURRENT CONTINUOUS CARE**					
Mean (SD)	1.43 (1.32)	1.03 (1.18)	1.59 (1.35)	*z* = 4.22	<0.001
**NUMBER OF SETTINGS DURING CURRENT CONTINUOUS CARE (*****n*** = **399**^a^**)**, ***n*** **(%)**					
2 settings	124 (31.1)	38 (32.8)	86 (30.4)	χ^2^ = 0.22	0.642
3 settings	90 (22.6)	14 (12.1)	76 (26.9)	χ^2^ = 10.30	0.001
>3 settings	73 (18.3)	15 (12.9)	58 (20.5)	χ^2^ = 3.15	0.076

*Mann–Whitney non-parametric test (z) used where continuous variables deviated from an approximately normal distribution*.

a *Data unavailable for 2 medium secure patients*.

A significant proportion of individuals did not remain in the setting to which they were originally admitted. On average, patients experienced 1.43 site changes in their pathway. The mean number of changes was significantly more for those currently in medium secure care than for those in high secure care. In terms of movement between settings, 31% had been in two sites, 23% in three sites, and 18% in four or more sites since their first admission to high or medium secure care. Many of these moves were from high to medium security but also from medium back up to high security. There was also a substantial amount of movement across the same level of security: 20% of the high secure sample had been admitted from another high secure setting, and 51% of those currently residing in medium secure care had come from another medium secure setting.

### Mental health act classifications

For the long-stay sample, the most common Mental Health Act (MHA) classification on admission to continuous secure care was section 37/41 (hospital order with restrictions; 22%), followed by section 3 (20%) and section 47/49 (transfer from prison to hospital with restrictions; 16%). The most common MHA classification on admission to the current unit was also section 37/41, for both high and medium secure samples. A number of patients experienced changes in their MHA section during their time in secure services; for example, the proportion of patients on section 37/41 on admission to their current unit was more than twice as great as that when admitted to continuous secure care (47.6 vs. 22.0%, χ^2^ = 57.95; *p* < 0.001).

A significantly larger proportion of patients in high secure care had initially been admitted on section 47/49 (24.1%) compared to those in medium secure care (12.7%; χ^2^ = 8.05, *p* = 0.005) reflecting findings regarding admission source to these two settings. This difference was also observed for admission to the current unit, where 20.7% of the high secure sample compared to 7.7% of the medium secure sample were admitted on a section 47/49 (χ^2^ = 13.66, *p* < 0.001). No statistically significant differences were observed between current high and medium secure patients in their current MHA section.

### Psychiatric treatment history

The mean age at first admission to any inpatient psychiatric service (secure or non-secure) in the overall sample was 22 years, with 67.8% (*n* = 272) of patients having had previous admissions to non-secure psychiatric inpatient care. The mean number of previous admissions was 4.3. Of particular note is the high number of patients with previous admissions (i.e., prior to the current continuous care episode in secure care that may in itself include admissions to a number of consecutive units): 46.4% (*n* = 183) had previous admissions to some level of secure psychiatric inpatient care. Few differences were found between our high and medium secure samples with regards to psychiatric treatment history, although those currently residing in high secure care had a higher percentage of previous high secure admissions (22.4% vs. 9.3%; χ^2^ = 12.39; *p* < 0.001).

Nearly two-thirds of the patients had a history of self-harm or suicidal behaviour, with no significant differences between the samples (**Table 5**). Overall, 35% of the long-stay sample had a history of serious suicide attempts; this figure was significantly higher in the high secure group compared with those currently residing in medium secure care.

### Current mental disorders

The most prevalent single diagnosis was schizophrenia at 57.9% (*n* = 232), with 32.8% of these patients considered to be treatment resistant. Personality disorder was the second most prevalent diagnosis (46.7%, *n* = 186), with antisocial PD the most prevalent type (68.3% of those with a PD diagnosis, *n* = 127) followed by borderline PD (46.2%, *n* = 86), paranoid PD (7.0%, *n* = 13) and narcissistic PD (5.4%, *n* = 10). Seventy three (39.7%) of those patients with PD had a mixed diagnosis of two or more PD types. Sixty five patients (16.2%) were recorded with a diagnosis of intellectual disability, and 12.8% (*n* = 51) had current alcohol or other substance misuse issues or dependence.

There were no statistically significant differences in broad primary diagnostic categories, although of patients diagnosed with PD a higher percentage of those in high secure care had antisocial PD (78.9 vs. 63.6%; χ^2^ = 4.32; *p* = 0.038) or two or more PD types (50.9 vs. 33.8%; χ^2^ = 4.83, *p* = 0.028). Intellectual disability was also higher in high secure care (24.1 vs. 13.3%; χ^2^ = 7.00, *p* = 0.008) which may reflect bed availability in medium and high secure care for these individuals.

### Offending history

The mean age at first conviction was 20 years. Most individuals (58%) in the long-stay sample were classed as primarily violent offenders. Although less than 6% were primarily sex offenders, 22% had committed both sexual and violent offences (Table 4). Twenty nine individuals (7.2%) had never been convicted of any offence. The scores for severity of offending were mainly in the mid-range (scores of 1 or 2). Excluding time on remand, 57% had previously had a custodial sentence. There were no differences between the high and medium secure groups in terms of any of these general descriptors of offending.

**Table 4:** Offending history of long-stay forensic patients.

	**Whole sample (*n* = 401)**	**High security (*n* = 116)**	**Medium security (*n* = 285)**	**Statistic**	***p*-value**
**CATEGORY OF OFFENDER**, ***n*** **(%)**					
Violent	232 (57.9)	72 (62.1)	160 (56.1)	χ^2^ = 1.19	0.276
Sexual	23 (5.7)	9 (7.8)	14 (4.9)	χ^2^ = 1.24	0.266
Mixed violent and sexual	88 (21.9)	21 (18.1)	67 (23.5)	χ^2^ = 1.41	0.236
Other	29 (7.2)	8 (6.9)	21 (7.4)	χ^2^ = 0.03	0.869
Non-offender	29 (7.2)	6 (5.2)	23 (8.1)	χ^2^ = 1.03	0.310
**SEVERITY OF OFFENCE (*****n*** = **364**^a^**)**, ***n*** **(%)**					
Score 0	107 (29.4)	26 (24.1)	81 (31.6)	χ^2^ = 2.10	0.148
Score 1	147 (40.4)	43 (39.8)	104 (40.6)	χ^2^ = 0.02	0.886
Score 2	77 (21.2)	27 (25.0)	50 (19.5)	χ^2^ = 1.36	0.243
Score 3	33 (9.1)	12 (11.1)	21 (8.2)	χ^2^ = 0.78	0.377
**TOTAL NUMBER OF OFFENCES (*****n*** = **395**^b^**)**					
Mean (SD)	15.28 (18.81)	18.28 (19.86)	14.03 (18.25)		
Median (range)	9.0 (0–130)	12.5 (0–118)	8.0 (0–130)	*z* = 2.52	0.012
Arson conviction, ever, *n* (%)	79 (19.7)	31 (26.7)	48 (16.8)	χ^2^ = 5.09	0.024
Custodial sentence, ever (*n* = 390^c^), *n* (%)	222 (56.9)	68 (59.1)	154 (56.0)	χ^2^ = 0.32	0.569
**AGE AT FIRST OFFENCE (*****n*** = **365)**					
Mean (SD)	19.99 (8.16)	19.30 (7.77)	20.29 (8.32)	*t* = 1.06	0.291
**TYPE OF INDEX OFFENCE**, ***n*** **(%)**^d^					
Offence against the person	232 (69.5)	70 (72.2)	162 (68.4)	χ^2^ = 0.47	0.493
Sex offence	78 (23.4)	16 (16.5)	62 (26.2)	χ^2^ = 3.59	0.058
Property offence	66 (19.8)	25 (25.8)	41 (17.3)	χ^2^ = 3.12	0.078
Theft and kindred offences	30 (9.0)	9 (9.3)	21 (8.9)	χ^2^ = 0.02	0.904
Fraud and kindred offences	1 (0.3)	0	1 (0.4)	χ^2^ = 0.41	0.522
Police/prison/court offence	6 (1.8)	2 (2.1)	4 (1.7)	χ^2^ = 0.06	0.815
Gun/offensive weapon offence	17 (5.1)	4 (4.1)	13 (5.5)	χ^2^ = 0.26	0.607
Other offence	15 (4.5)	3 (3.1)	12 (5.1)	χ^2^ = 0.62	0.430
Having no index offence, *n* (%)	66 (16.5)	19 (16.4)	47 (16.5)	χ^2^ = 0.00	0.978

*Mann–Whitney non-parametric test (z) used where continuous variables deviated from an approximately normal distribution*.

a *Data missing from 8 high secure and 29 medium secure patients*.

b *Data missing from 6 medium secure patients*.

c *Data missing from 1 high secure and 10 medium secure patients*.

d *As percentage of those with an index offence*.

Those currently in high secure care had a significantly higher mean total number of offences (18.3 vs. 14.0). In terms of number of particular offences, those in high secure care had higher mean numbers of offences against the person (4.8 vs. 2.7; *z* = 2.58; *p* = 0.010) and property offences (4.1 vs. 2.3; *z* = 2.74; *p* = 0.006) but no differences were found for any of the other Police National Computer offence categories. Seventy nine (20%) of the long-stay sample had convictions for arson; patients with an arson conviction were more prevalent in the high secure group.

A total of 66 patients (16.5%) did not have an index offence prompting admission. Of those with an index offence, this was most commonly an offence against the person; a sexual offence was the second most common category (Table 4). For those with a violent index offence, homicide was the most common category; for those with a sexual index offence, indecent assault was the most common, followed by rape or attempted rape. There were no significant differences in any of the index offence variables between current high and medium secure patients with the exception of attempted rape which was more common in the medium secure sample (χ^2^ = 4.40, *p* = 0.036).

### Institutional behaviour

A large number of individuals in this long-stay sample had convictions for violent or sexual offences in institutional settings (27%), with significantly higher figures for high secure care. 26% had committed a serious assault on staff in the last 5 years (Table 5). A significant proportion of patients had at some point been involved in serious incidents in an institutional setting, such as absconding, room barricade, attempted hostage taking, rooftop protest, or rioting. Twelve percent had seriously self-harmed (requiring medical attention) and 44% had been in seclusion during the past 5 years. Incident indices were significantly higher in current high secure patients, including successful room barricade (16 vs. 8%), serious assaults on staff in last 5 years (42 vs. 19%) and seclusion episodes in last 5 years (68 vs. 35%).

**Table 5:** Institutional behaviour of long-stay forensic patients.

	**Whole sample (*n* = 401)**	**High security (*n* = 116)**	**Medium security (*n* = 285)**	**Statistic**	***p*-value**
**SERIOUS INCIDENTS, EVER**, ***n*** **(%)**					
Conviction for violent/sexual offence while in an institutional setting	108 (26.9)	48 (41.4)	60 (21.1)	χ^2^ = 17.31	<0.001
History of self-harm or suicidal behaviour	256 (63.8)	81 (69.8)	175 (61.4)	χ^2^ = 2.53	0.111
History of serious suicide attempt(s) (*n* = 399^a^)	141 (35.3)	53 (46.1)	88 (31.0)	χ^2^ = 8.17	0.004
Successful absconsion, ever (*n* = 399^b^)	159 (39.8)	43 (37.1)	116 (41.0)	χ^2^ = 0.53	0.468
Attempted hostage taking	17 (4.2)	6 (5.2)	11 (3.9)	χ^2^ = 0.35	0.554
Successful room barricade	41 (10.2)	19 (16.1)	22 (7.7)	χ^2^ = 6.74	0.009
Successful rooftop protest	6 (1.5)	2 (1.7)	4 (1.4)	χ^2^ = 0.06	0.811
Involved in a riot	5 (1.2)	2 (1.7)	3 (1.1)	χ^2^ = 0.30	0.583
**SERIOUS INCIDENTS/SECLUSIONS IN THE LAST 5 YEARS (*****n*** = **397**^c^**)**, ***n*** **(%)**					
Serious assault on staff	102 (25.7)	48 (42.1)	54 (19.1)	χ^2^ = 22.56	<0.001
Serious assaults on others	110 (27.7)	38 (33.3)	72 (25.4)	χ^2^ = 2.53	0.112
Serious self-harm	46 (11.6)	18 (15.8)	28 (9.9)	χ^2^ = 2.76	0.097
Seclusion episode(s)	176 (44.3)	77 (67.5)	99 (35.0)	χ^2^ = 34.91	<0.001

a *Data missing from 1 high secure and 1 medium secure patient*.

b *Data missing from 2 medium secure patients*.

c *Data missing from 2 high secure and 2 medium secure patients*.

### Current management and treatment

In terms of diagnostic specification, 42.6% (*n* = 171) of the long-stay patients resided on a mental illness ward at the time of data collection; other ward diagnostic specifications were “personality disorder” (13.2%, *n* = 53), “intellectually disability” (11.5%, *n* = 46), “comorbidity” (10.0%, *n* = 40), “neuropsychiatry” (4.7%, *n* = 19), and “mixed” (17.5%, *n* = 70). In total, 51.1% of the sample (*n* = 205) were currently receiving some form of psychological treatment. Where treatment modality was specified, cognitive-behavioural interventions were by far the most frequently mentioned, followed by dialectical-behavioural therapy. Despite the high risk that the long-stay sample presents, only a relatively small proportion of patients currently in high secure care were on telephone or mail monitoring at the time of the study (12.9% and 20.7%, respectively).

### Referrals and tribunals

Patients had an average of 2.23 (SD 1.05) tribunals in the past 5 years with no significant differences between groups, suggesting probably a mix of automatic referrals (every 3 years) and patient applications. An unsuccessful referral to a setting of lower security was recorded over the last 5 years for 95 patients comprising 32.9% of the sample, with no significant differences between the groups.

## Discussion

### Main findings

This paper reports on findings from a large multicentre study and provides the first representative description of long-stay forensic patients in England. Data was obtained from file reviews from a sample of 401 patients from two high secure settings and from 23 medium secure settings on 1 April 2013.

As is often found in general forensic samples, the sociodemographic characteristics of this long-stay sample are suggestive of early disruptive lives with patients not having achieved stable relationships or employment, and are broadly comparable with those reported in a recent survey of patients in long-term forensic psychiatric care in the Netherlands [25]. Of particular note is that two thirds of the current sample had no formal qualifications before admission, which indicates that many patients who stay for extended periods in forensic psychiatric care have significant educational needs. Addressing these needs can mitigate many of the difficulties faced by these patients when finally discharged, and an extended period of hospitalization offers the potential for improving educational skills. However, in this study insufficient data were collected to allow any conclusion to be drawn on the nature of any educational opportunities offered to these inpatients during their stay.

Perhaps unexpectedly, the majority of patients had some form of contact with their families and/or others outside the secure setting. It is not clear whether this is due to staying in or renewing contact with families; clinical experience suggests that the latter contributes a significant proportion of family contact, but further research is required to confirm this. Whilst it is unclear how supportive these relationships are, such contact can be a significant protective factor against violence risk [26] and this finding is therefore relevant when planning for patients' recovery. It also places some responsibility on services to support carers and maximise the opportunities for meaningful interactions between patients and their families.

As expected, the most prevalent diagnosis was schizophrenia followed by PD. Unlike studies of general UK forensic populations which found rates of PD of about one-third in medium security [27, 28] and 45% in high secure care [29], findings from this long-stay sample suggest higher rates of personality pathology in both levels of security. For those who remain in secure care, personality pathology is likely to present a significant treatment need, possibly because psychotic illness abates rapidly with anti-psychotic treatment leaving individuals with damaged personalities who are often challenging and difficult to treat, and because personality dysfunction is likely to impact on other areas of functioning such as relationships, motivation and engagement [30]. The finding that individuals with intellectual disabilities form a significant proportion (16%) of this long-stay sample should be interpreted cautiously owing to the deliberate oversampling of units catering to this group, although this figure is comparable with, for example, the proportion (19%) reported in a recent Canadian study [31]. Findings from other studies in the UK [32] and in Canada [33], for example, on the impact of intellectual disabilities on length of stay are inconsistent. It is nonetheless worth noting that those with intellectual disabilities in high secure settings have been reported to have a larger number of unmet needs than other patient groups and so may not be able to move on because of a lack of facilities in less secure settings [34]. This issue may be compounded by recent initiatives to close down institutions for patients with intellectual disabilities [35].

Just under two-thirds of this long-stay sample were primarily violent offenders, in keeping with other research in the UK [12], in Sweden [36], and in Ireland [6]. The prevalence of a sexual offence and arson as index offences appear to be higher than those reported in the general forensic population which could suggest a lack of effectiveness of interventions offered to these offenders, or difficulties with moving such offenders on, or both. Two apparently anomalous findings require consideration. These are that 7.2% of the sample had never been convicted of any offence, and that 16.5% did not have an index offence prompting admission. These situations may appear to raise ethical issues, particularly as in many countries such patients could not be legally admitted to secure care even though such a placement may best reflect their needs. As already noted, offending is not a prerequisite for entry into forensic-psychiatric services in the UK, and patients admitted to UK secure institutions without a formal history of offending are often those who present with challenging behaviour in general psychiatric services, making it impossible to safely care for them there. It is also relevant that, as in other European countries, prisoners in England and Wales who develop mental illnesses in prison can be transferred to forensic psychiatric facilities when their disorder warrants it [37].

Recent behaviour within institutions might arguably be at least as important as previous offending in determining future placement, in particular for those whose index offences are many years in the past. The current study has revealed high numbers of patients involved in incidents within institutions, including serious assaults on staff, seclusion episodes, and convictions for violent and sexual offences. In terms of the prevalence of violent assault toward patients, these findings are comparable with those estimated in a recent study of forensic psychiatric patients in North America [38], although the estimated prevalence of 16% for violence toward staff in that study is somewhat less than the figure of 26% reported here for serious assault on staff in the last 5 years. This suggests that a significant proportion of long-stay patients remain unsettled and are therefore likely to require high staffing levels and measures for behavioural management, such as access to seclusion facilities, in any future setting. There is, however, also a group that has not engaged in intra-institutional behavioural disturbance, and these patients might be manageable in a less highly staffed environment.

In terms of MHA classification, it is relevant that the majority of patients in both high and medium secure long-stay samples were detained under section 37/41. The section 41 restriction requires that permission must be obtained from the Ministry of Justice before a patient can be conditionally discharged from hospital or transferred to another, e.g., less secure, setting. Such permission may not be forthcoming or may be delayed, and so section 41 may act as an obstacle to moving on and, arguably, result in a longer stay. Interestingly, the proportion of patients on section 37/41 on admission to their current unit was more than twice as great as that when originally admitted to continuous secure care. This suggests that, over time, patients move to a situation in which their legal position makes any positive moves more difficult to achieve. These sections may also reflect ongoing psychopathology and/or offending within secure settings which will also result in longer stays.

Patients in this long-stay sample primarily entered the forensic psychiatric system via prison, which is in line with studies on general forensic populations [35]. A different pattern emerges when entry to their current unit is considered, however, with significantly fewer admitted from prison and correspondingly more admitted from other secure settings. This suggest that a significant proportion of individuals not remain in the setting to which they were originally admitted, and that their pathways are complex. Only a minority experienced no moves or only one move along the ideal treatment pathway, i.e., from higher to less secure settings. The direction of these moves is particularly important. Some are moves upward in security, presumably triggered by worsening symptoms or increased behavioural disturbance. Some are moves downward in security, presumably as a result of a more settled presentation and some progress toward recovery. However, the significant percentage of patients making sideward moves suggests that the ideal pathway of moving from higher to lower levels of security is, in reality, not achieved for most patients. This situation is further compounded because a significant proportion (about one third) of this long-stay sample experience unsuccessful referrals to less secure settings, in some cases repeatedly.

The reasons for these moves between settings at the same security level are unclear and warrant further investigation. One possibility concerns the commissioning of medium secure beds in the independent sector. Beds that are purchased on an individual basis may be scrutinised more closely than those purchased on a block contract basis leading to shorter placements [39]. Another possibility is that some are the result of so-called “repatriation” from out-of-area placements to patients' home areas, which on the one hand might facilitate contact with family or friends but on the other hand may lead to disruption of treatment. A number of reasons may be suggested as to why patients remain “stuck” at a particular level of security [28, 40, 41]. These include inconsistencies in criteria applied to moving to less secure settings, differences in opinions between consultants in different services, and delays in the assessment and transfer process. Various suggestions have been made to improve this system, including paper-based assessments, single assessments, and appeal panels [30].

### Limitations

This study provides a national picture of long-stay in both NHS and independent forensic settings and considers whole pathways rather than just admission to single units. Several important limitations can, however, be identified. First, although stratified sampling was used based on geographical location and size, not all available units contributed to the study which may limit representativeness of the sample. Second, the oversampling of units catering for female patients and those with intellectual disabilities may have led to some overestimation of the prevalence of patients with these characteristics. Third, the detailed file reviews were conducted by local collaborators rather than by the study's research staff; using the latter may have resulted in more consistent data recording even though attempts were made to maximise consistency by through training exercises and regular communication with data collectors. Fourth, the nature of the study means that the results may not be generalizable outside the UK.

### Implications for research

First, further research is needed to investigate in more detail the impact family contact might have on patients' progress. Second, since there was a significant proportion of this long-stay sample for whom referral to a less secure setting was unsuccessful, a closer inspection of these cases might reveal unmet service needs. Third, further research into the treatment pathways of longer stay patients is clearly warranted. The complexity of these pathways is striking in this long-stay sample, and is likely to be confusing for (and frustrating to) patients and carers, as well as inefficient and costly. It would be helpful to compare pathways, staffing levels and outcomes of general forensic care in other European countries to identify why some countries are able to provide forensic care that is less resource intensive [42].The current study has shown that a whole life-span view is needed to understand the complex trajectories of this group.

In addition, any future study might also usefully examine any change in patients' diagnoses whilst in care and the prevalence of ADHD and dyslexia diagnoses in long-stay samples, particularly as there is growing evidence that ADHD diagnoses are over-represented in young violent offenders [43] and in forensic psychiatric patients [44, 45], and that dyslexia and ADHD can be prevalent in forensic patients convicted of sexual offences [46]. It would also be useful to explore any relationship between benzodiazepine use and violent or suicidal behaviour, given that benzodiazepines can be associated with increased suicide risk [47], with paradoxical aggressive reactions [48, 49] and with violent behaviour in forensic patients [50, 51].

### Implications for practice

Although this study has identified some differences between the high and medium secure samples, the long-stay groups show more similarities than differences across settings. This raises a question concerning the reliability of allocation to one or the other setting. One conclusion is that the long-stay group should be treated as a separate category outside the medium and high secure categorisation. Changes to the care of these patients, involving potentially quicker throughput or step-down, could lead to substantial savings as well as improvements in the patients' quality of life. Evidence from this detailed file review suggests that interventions offered have not resulted in sufficient changes to allow these patients to move on, and the distinction between high and medium secure care does not appear to be fully applicable to this group. Consideration should be given to the development of a separate long-stay service as available in other countries with positive effects on quality of life of patients. This form of service could be configured to provide care for two contrasting groups of long-stay patients: those whose behaviour continues to be very challenging, and those who are not regularly engaged in intra-institutional behavioural disturbance and who might be manageable in a less highly staffed environment.

## Author's note

This article is based on a study first reported as Völlm B, Edworthy R, Holley J, Talbot E, Majid S, Duggan C, et al. A mixed-methods study exploring the characteristics and needs of long-stay patients in high and medium secure settings in England: implications for service organisation. *Health Serv Deliv Res* 2017; 5:11.

## Author contributions

BV: conception and design of the project, analysis and interpretation of data, manuscript drafting and revision; RE, ET, and SM: design of the project, data acquisition, data entry, interpretation of data, manuscript drafting and revision; NH: analysis and interpretation of data, manuscript drafting and revision; JH: design of the project, interpretation of data, manuscript drafting and revision; VF: conception and design of the project, data acquisition, analysis and interpretation of data, manuscript drafting and revision; TW, RM, and CD: conception and design of the project, interpretation of data, manuscript drafting and revision.

### Conflict of interest statement

The authors declare that the research was conducted in the absence of any commercial or financial relationships that could be construed as a potential conflict of interest.
